# Chemical Experiments and Observations, on the Extraction of Sugar and Syrup from Indigenous Plants

**Published:** 1800-09

**Authors:** Sigism. Henry Hermbstaedt


### *Chemical Experiments and Obſervations, on the Extraction of Sugar and Syrup from Indigenous Plants.* By SIGISM. HENRY HERMBSTAEDT.

FROM the chemical analyſis of vegetable ſubſtances, and the knowledge of their conſtituent and other particles contained and mixed with them, it is ſufficiently evident, that the Eaſt and Weſt Indies are not the only countries provided by Nature with ſaccharine plants, but ſaccharine matter is abundantly found in other productions of the vegetable kingdom; and it only requires an aſſiduous examination to point out thoſe vegetables, from which it may be moſt copiouſly, and in the leaſt expenſive way, obtained.

Among the plants hitherto examined, none deſerve to be ranked ſo near the true ſugar-cane, as the whole genus of maple trees; and, particularly, the ſugar and ſilver maple, *Acer ſaccharinum*, and *A. daſycarpon ehrh.* Both trees have been uſed for theſe fifty years to obtain ſugar from,* which in the laſt eight years has proved to be exceedingly profitable.† By my

* An account of a ſort of ſugar made of the juice of maple in Canada; Philoſophical Tranſactions, No. 171. Klam, in the Tranſactions of the Royal Swediſh Academy of Sciences, year 1751. Memoires ſur les ſucre d'Erable uſité dans le Canada, &c. Hiſt. 1757.

† Notices ſur l'Erable à ſucre des états unis, et ſur les moyens d'en extraire le ſucre, &c. par M. Ruſh, in Rozier's Obſervations ſur la Phyſique, &c. Tom. xli. Paris, 1792.

my own experiments, which I have repeatedly made fince the winter of 1796, I found out, that from all fpecies of maples, fugar may be with more or lefs profit obtained, and that the fugar and filver maple growing even in Germany, though not in the beft foil, give a very good raw fugar, not inferior to the beft Weft India cane-fugar, and which is got fo cheap, that a pound of it will come no higher than eighteen or twenty plennige, or about two-pence halfpenny; and only a grofhen, or a penny, when, inftead of charcoal, common coal or turf are employed for boiling the juice, and particularly when the operation is made upon a large fcale, as one labourer is able to attend 500 trees during the period of tapping them. The procefs of boiling the juice is, befides, fo very fimple, that any perfon may foon learn it. But thefe advantages are only to be expected from the fugar and filver maple, as the other fpecies, *Acer negundo*, *A. campeftre*, *A. platanoides*, and A. pfeudoplatanus contain a lefs quantity of fugar, which is alfo not fo rich in faccharine matter. However, as plantations of thofe maples require a fpace of twenty or twenty-five years before the trees are large enough to admit tapping, it will not be improper, but of great utility to the community, to examine in the mean while thofe indigenous plants, from which likewife a ufeful fubftitute for the Weft India fugar may be extracted; and it is with this view I have made the following experiments.

### 1. *Experiments to obtain Sugar from India Corn.*

India corn, (zea mays) is faid to contain, according to Vonfufti,‡ fugar, particularly in the nodes of the young ftalks, from which Mr. Jacquin of Vienna,§ has fuccefsfully prepared it; and this is farther confirmed by Mr. Marabelli ‖ in a differtation on the fubject. It is likewife reported, that the extraction of fugar from the ftalks of India corn, growing particularly in marfhy foils, has been tried in Italy upon a large fcale, but afterwards left off again, as it was found not to anfwer the purpofe, the fugar thus obtained being more expenfive than common raw fugar. To be convinced by my own experience on this fubject, I made fome experiments, of which the following are the refults: A quantity of India corn was cultivated, in a tolerable and fomewhat marfhy foil, for the purpofe. When the young plants were about fix inches high, the

‡ Okonomifche Schriften, i. e. Economical Writings, Tom i. p. 307, and Tom ii. p. 191.

§ Crell's Chemical Annals, 1784, Vol. I.

‖ Franc. Marabelli de Zea Mays planta, analytica difquifitio. Pavia, 1793.

the leaves, when chewed, had a ſweetiſh taſte, but the ſtalks, particularly about the nodes, taſted like ſugar. Theſe young plants being cut off as near the ground as poſſible, freed from the leaves, and ſufficiently cleaned, ten pounds of them were cut in pieces, and being pounded in a ſtone mortar, the juice was expreſſed, which weighed three pounds. This juice, whoſe ſweetiſh taſte had ſtill a diſagreeable flavour of herbs, was clarified with the white of eggs, after which that taſte was ſcarcely perceptible, and being thickened to the conſiſtence of a ſyrup, eight ounces of a very agreeable taſting ſyrup were obtained.

2. *Examination of the Spikes of India Corn.*

As the young ſpikes, when they are beginning to form, poſſeſs a very agreeable ſaccharine taſte, they were thought fit for being examined. Ten pounds of them were accordingly ſqueezed in a ſtone mortar, and the juice expreſſed, after the leaves had been ſtripped off. Theſe gave four pounds of a milky juice, which could not be rendered perfectly clear by the white of eggs. By a ſlow evaporation, to the conſiſtence of a ſyrup, nine ounces of an agreeable taſting brown ſyrup were got, but which differed from the former by being more mucilaginous.

3. *Examination of Stalks of India Corn of a more advanced Growth.*

Twenty pounds of theſe ſtalks were cut in pieces, and, with the addition of water, ſqueezed in a ſtone mortar and the juice expreſſed, which poſſeſſed a diſagreeable and ſomewhat acrid taſte. Being in the ſame manner clarified, and thickened to the conſiſtence of a ſyrup, twelve ounces of ſyrup were obtained, which had a diſagreeable ſaline taſte, and might rather be conſidered as a vegetable extract than as ſugar.

4. *Experiments for obtaining dry Sugar from India Corn.*

To learn whether it was poſſible to exhibit a cryſtallizable ſugar from this plant, the ſyrups prepared from the young ſtalks and the ſpikes were each diſſolved by itſelf in freſh lime-water, and gently boiled, by which a great part of their impurities was carried off. The liquors being ſtrained through a woollen cloth, each of them was boiled to the thickneſs of a ſyrup, which was put in a glaſs, and ſet for eight months in a warm place, when little cryſtallizations of ſugar appeared, which were with difficulty ſeparated from the fluid. For this purpoſe, each ſyrup was evaporated by a gentle fire till it became dry, and this maſs was digeſted with alkoholized ſpiritus vini to ebullition.

ebullition. The fluid, ftill hot, was inftantly poured through fome linen, whereon the mucilaginous parts remained; but on cooling of the fpiritous folution, a true fugar, of a yellow colour, cryftallized in fmall grains. The alkohol being drawn from the remaining fluid by diftillation, another portion of fugar was got by gentle evaporation, and altogether two ounces from the fyrup of the young ftalks, and one ounce and a half from that of the fpikes.

By thefe experiments it is fufficiently fhown, that from the young frefh ftalks, as well as from the fpikes of India corn, a true fugar can be extracted; but as its feparation from the gummy and other particles mixed with it, is attended with fuch difficulties, and the gain fo very inconfiderable, that a pound of raw fugar from this plant would coft one rix-dollar, or above three fhillings, it appears that no profit or economy will arife from the fabrication of this fugar.

5. *Experiments for obtaining Sugar from the Siberia Cow-parfnep.*

The Ruffian cow-parfnep, (*Heracleum fphondylium Linn. Heracleum Sibericum)* has been long known as a plant containing a great deal of faccharine matter, in which refpect, according to Steller, (in his Travels to Kamtfchatka, in German) it deferves the next place to the fugar-cane, and the natives call it therefore *sweet-herb*, or *katfh*. According to Gmelin (Flora Siberica, tom 1, p. 214.) it does not differ from our common cow-parfnep; but others think it a particular fpecies, to which they give the name of *fphondylium panaces*. The inhabitants of Kamtfchatka gather the ftalks of this plant in June, and having ftripped off the leaves, they fhave off the outer fkin with mufcle fhells, and dry them in the fun, and afterwards they are chewed for the fake of fucking out the faccharine matter. In drying, the furface of the ftalks is covered with a white faccharine powder, which they feparate by fhaking them in a leather bag; but 40 ℔. of them afford only a quarter of a pound of this powder fugar, which therefore is confidered as a great rarity. Befides this, the ftalks and roots of the plants are employed for obtaining a fort of brandy.

I was fupplied with fome frefh plants of the Heracleum Sibericum for my experiments; but finding that the ftalks were by no means fo rich in fugar, as it is related of thofe plants growing in Siberia, I tried the roots, of which I got four pounds, whofe tafte is fweetifh like that of parfneps. Having freed them from the outer fkin, they were dried, but no faccharine cruft appeared on the furface; they were therefore ground, and being mixed with water, the juice was expreffed, which tafted fweetifh but a little acrid. Being boiled with the white of eggs, and clarified, it was thickened to the confiftence of a fyrup, of which fix ounces were got, wherein, after a fpace of three months, a brown, grainy fugar had cryftallized, which, however, was not quite free from a difagreeable flavour.—Though it is fhown by thefe experiments, that fugar may be obtained from that plant, yet the preparation of the fugar is too expenfive for making ufe of it. It is, however, probable that the foil has a great influence upon the plant, and that therefore thofe growing in Siberia are richer in fugar.

### 6. *Experiments to obtain Sugar from the Muft of Wine.*

It might be prefumed from the tafte of muft obtained from ripe grapes, that a confiderable quantity of faccharine matter is contained in it, though involved by mucilage. To try whether a true fugar could be extracted from it, fome experiments were undertaken. Eight Berlin quarts of muft from ripe fweet grapes, were feethed with the white of eggs, clarified, and filtered. The fluid being evaporated, gave three pounds of an agreeable but acidulous fyrup. To take away this free acid, the fyrup was diffolved again in lime water, and fo much of it added, till no acid was perceived by reagency. The fluid being again clarified and evaporated, a very agreeable fyrup was obtained, but from which it was by no means poffible to procure cryftallized fugar. However, this fyrup would not be very profitable at the high price of muft.

### 7. *Experiments with the Juice of the white and black Birch for the fake of obtaining Sugar.*

It has been much doubted, whether fugar might be got from the juice of thofe trees. Mr. Staehlhammer, (Tranfactions of the Swedifh Academy, Vol. xxxv.) prepared from the juice of the white birch, *Betula alba*, tapped in the fpring, a confiderable quantity of fyrup, fuperior to the common brown fyrup, but not fo good as that from the maple. Mr. Kalm (*Ibid.* Vol. xiii.) relates, that from the North American black birch (*Betuli nigra, carpinifolia*), alfo called fugar birch, a great deal of fugar is obtained, which is not fo fweet as that from the maple. Having received a fufficient quantity of the juice of both trees, I found that from one hundred and fifty Berlin quarts of the juice, extracted in April from fifty trees of the white birch, two pounds and a half of brown fyrup were obtained, out of which no cryftallized fugar could be got. But fifty quarts of the juice of the black birch gave one pound and a half of a very good and ufeful fyrup, yet inferior to that of the maple. From this fyrup a confiderable portion of cryftallized fugar feparated; parated; by which it appears, that ſugar may be obtained from the black birch, but much inferior in quality to that of the maple, and, at the ſame time, more expenſive.

8. *Experiments to obtain Sugar from the Beet. (Beta eicla alba.)*

It is more than fifty years ſince the late Mr. Marggraff made ſeveral experiments with different ſpecies of the beet, to try how far it was poſſible to obtain a uſeful ſugar and ſyrup from them; in which, however, he ſeems to have particularly ſucceeded with *Beta eicla*, as he extracted from 2 lb. of its freſh roots, half an ounce of good ſugar. Induced by theſe experiments, I took 68 lb. of the roots of beet, which being ſufficiently cleaned, were ground and ſqueezed, and the reſiduum being moiſtened with warm water and again expreſſed, about 20 quarts of juice were obtained. A third part of this having evaporated, it was then mixed with 20 quarts of lime-water, and boiled for half an hour. The juice thus become clear, and of a yellowiſh colour, was filtered, and afterwards thickened to the conſiſtence of a ſyrup, of which 6 lb. were obtained in this way, of a brown yellowiſh colour, and an agreeable taſte. A part of it was poured into an evaporating glaſs diſh, in which ſome glaſs tubes had been placed; and being expoſed for ſixteen weeks in a warm ſituation, thoſe tubes were found to be incruſted with cryſtals of ſugar, about the ſize of a pea, and very much like brown ſugar-candy. A buſhel of the beet roots coſts about half a crown, and the expence of obtaining a ſyrup from them is not much above ſix-pence; a pound of the beet ſyrup would, therefore, come no higher than about four-pence, which is leſs than the price of the common ſyrup in Germany; conſequently, it might prove a very advantageous ſubſtitute. The ſugar may likewiſe be obtained cheap enough, in compariſon to the preſent high price of the cane-ſugar; but the operation to extract it is very long and ſlow; and though it equals the ſugar of the maple in goodneſs, yet the proceſs of extracting it is far more expenſive.

9. *Experiments to obtain Sugar from another Species of Beet, (Beta vulgaris altiſſima, or Beta eicla altiſſima Jacquin. — Bunkelrube, Germ.)*

This ſpecies of beet-root is commonly cultivated in ſome parts of Germany, as a very good food for cattle, and differs from the former by being more ſucculent and of a ſweeter taſte. The roots grow larger, and contain more juice, when cultivated in a rich ſoil. A buſhel of theſe roots, which weighed about 125lb. after the outer rind had been taken off, were ground ground upon a grater; and after a part of the juice had discharged itself during the grinding, the rest was expressed; by which means, 24 quarts of a very sweet violet-coloured juice were obtained. Taught by repeated experiments, I first boiled it to the evaporation of one-third, by which a great deal of albuminous matter separated. When cool, it was poured through a woollen cloth, mixed with 24 quarts of lime-water, and boiled for half an hour. The juice became in this way a clear fluid of a yellowish colour, being freed from a great part of its impurities. After having cooled and strained it, it was boiled to the thickness of a syrup, of which eight pounds were got. A part of this was likewise exposed in a deep evaporating dish, in which glass tubes were put, to a gentle evaporation, and after two months a deep yellow sugar was found to have crystallized on those tubes. As the crystallization of sugar proceeds very slowly in this syrup, as well as in the former, the quantity of sugar which it may contain cannot be determined; but as this syrup is by far more agreeable than common syrup, and moreover as, including all expences, a pound will cost no more than about three halfpence, it may likewise serve as a useful substitute. This species of beet is the same which Mr. Achard, of Berlin, has since used for the fabrication of sugar upon a larger scale.

10. *Experiments for obtaining Sugar from the red Beet, (Beta rubra, Beta eicla rubra, Jacquin.)*

The possibility of obtaining sugar from the common red beet has been already shown by Marggraff; I proceeded therefore in the same way as with the former, and obtained from a bushel of its roots six pounds and a half of syrup, which had rather an unpleasant rough taste, from which, however, it may perhaps be freed. I did not try to get sugar from it, though Marggraff obtained from 8 ounces of dried roots 2½ drachms of sugar.

[ To be continued. ]

